# Mr. Douglas's Case of Convulsions

**Published:** 1807-06

**Authors:** J. C. Douglas

**Affiliations:** Surgeon, Tipperary Militia. Dublin


### *To the Editors of the Medical and Physical Journal.*

Gentlemen,

In the early part of June last, about seven o'clock P. M. I was called to see Mrs. H. a person in comfortable circumstances of life, of a full habit of body, of a temperament rather irritable, and commencing her last month of pregnancy.

From noon of the same day she had been somewhat indisposed, and had progressively gotten worse towards evening; she was at this time affected with drowsiness approaching to stupor, her eyes had a heavy and at the same time a wild appearance, and I would imagine there was incorrectness of vision. Her answers to any questions proposed, were uttered with seeming indifference and much incoherence, denoting considerable vacillation of mind. She also occasionally fetched a deep sigh. In short, there were symptoms sufficient to have warned a more experienced practitioner of the impending danger; but not having myself seen many such cases, and forming a hasty opinion in this instance without much enquiry, observation or reflection, I concluded that the lady had taken rather ther freely of exhilarating fluids, a custom to which, I am now well convinced, she was by no means addicted, but which conclusion I at the moment drew, not only from the incorrectness of her words and actions, but also from the appearance of her countenance, which was highly flushed, another forerunner of the approaching attack, but which I attributed to the cause assigned, assisted by the influence of a large fire, near to which she sat.

From the opinion I formed, I did not think it necessary to order any medicine except merely for appearance sake, I therefore directed that she might be put to bed, that the fire in the room might be lessened, and that she might get a dose of castor oil or have an injection thrown up the rectum. I then took my leave without feeling further concern for my patient, until after the lapse of two hours, when a messenger came to inform me that she was just dying; much surprized, I hastened to her house, which was only a few doors from my lodgings. I found her in the following state:—The entire of her head appeared very considerably swelled, every feature enlarged and distorted, the colour of her face, instead of being florid as when I had first seen her was now nearly black, fully as much so as an African's, her lips also had the appearance of a person of that colour, with respect to breadth and thickness; her breathing was difficult and stertorous, and her entire frame was in some degree affected with convulsive motions. Although I had not, at my first visit, either committed any direct error as to practice, or exposed my ignorance of the complaint, as what directions I gave were given *secundum artem,* yet I could not avoid feeling much chagrined at not having a conception of the formidable attack which had been impending. I immediately called for further assistance; in the mean time I could not venture to delay making use of the most immediate means of relief, lest I might thereby lessen the chance of her recovery, an event which I thought very precarious. I therefore, as quickly as possible, opened a vein in the arm, and when about twenty ounces of blood had issued, I could perceive that the breathing became somewhat easier, and that the distortion and enlargement of the features were beginning to subside; finding all the untoward symptoms still further to abate as the blood flowed, I allowed it to go on until not less than forty ounces had come away, the pulse still continuing above par, both as to fulness and strength.—I now however tied up the arm; after which Mr. Middleton, a respectable practitioner in Mullingar, where where the case occurred, entered the room, and while he was feeling the pulse and observing other symptoms, I mentioned that I had taken about five half pints of blood; he thinking that I had said half a pint, advised more to be taken, as the pulse would bear it; but when he saw the quantity he seemed to think it quite sufficient. I mention this circumstance to shew that I was not mistaken with respect to the state of the pulse.

The dread of immediate danger being now averted, our attention was to be directed to the prevention, if possible, of a recurrence of the convulsions, as well as to repair the injury which the system had sustained. In such violent derangement as the present, little effect could be expected from the castor oil which she had gotten in the first instance, therefore a very stimulating injection was administered, which produced a copious evacuation; and with a view to rouse her from a comatose state, a blister was applied between her shoulders. The stupor went off in a few hours, and next morning she appeared tolerably well. She was then ordered some purgative medicine, (a strong infusion of senna with tincture of jalap,) of which she was obliged to take large and repeated doses before it operated. On the third morning she was perfectly able to sit up in her bed, no symptom of disease remaining. As yet no appearance of labour could be perceived; however, on the fourth evening it commenced, and after three or four hours of easy labour, she brought forth a living child. She recovered rapidly and is now nursing her child.

Although I am persuaded that my time would be better employed in reading than in writing, yet having committed myself so far, (in which I have been partly actuated by a desire to put other inexperienced practitioners on their guard, when called to patients in a state similar to that in which I found this lady on my first visit,) I will venture to offer a few observations on the preceding case. And first to observe,

That I have no doubt, if I had bled largely, perhaps to the amount of a pound and a half on my first visit, no convulsions would have occurred. It may be asked—Might not a more moderate bleeding have answered the purpose of relieving this woman? Although the immediate danger was perhaps averted when little more than half the quantity had been taken, yet I am well satisfied, that if I had stopped then, there would in all probability have have been a recurrence of the convulsions, which might have proved fatal to the mother or child, or both.

There was another circumstance with those already mentioned, which appeared to me, to indicate the necessity, or at least the propriety, of continuing the evacuation to the extent I did. The velocity with which the blood issued from the orifice of the vein, which was much of the common size; for, as by the laws of Hydrostatics, the velocity with which a fluid spouts out at a hole in the side or bottom of a vessel is directly as the height of the fluid above that hole, and consequently as its pressure; so did I conceive, from the velocity of the flow, that there must have been great pressure on the blood at the orifice of the vein; not as in the other case, from the pressure of the superincumbent fluid, but from an increase of irritability and the violence of vascular actions, which were thus to be subdued.

It were much to be wished that practitioners of authority, when speaking of blood-letting in particular diseases requiring great evacuation, would sometimes, instead of using the words great, plentiful, copious, mention the greatest extent, particularly in ounces, to which they had carried it either with advantage or impunity, as a guide to young practitioners, such as the writer of this paper.

I am, &c.

*Dublin,* J. C. DOUGLAS, Surgeon,

*January, 23, 1807.* Tipperary Militia.

